# Efficacy of Typhoid Conjugate Vaccine in Nepal: An Interim Analysis of a Participant-and Observer-Blinded Randomized Phase III Trial

**DOI:** 10.1056/NEJMoa1905047

**Published:** 2019-12-05

**Authors:** Mila Shakya, Rachel Colin-Jones, Katherine Theiss-Nyland, Merryn Voysey, Dikshya Pant, Nicola Smith, Xinxue Liu, Susan Tonks, Olga Mazur, Yama G. Farooq, Jenny Clarke, Jennifer Hill, Anup Adhikari, Sabina Dongol, Abhilasha Karkey, Binod Bajracharya, Sarah Kelly, Meeru Gurung, Stephen Baker, Kathleen M. Neuzil, Shrijana Shrestha, Buddha Basnyat, Andrew J. Pollard

**Affiliations:** a Oxford University Clinical Research Unit, Patan Academy of Health Sciences, Kathmandu, Nepal; b Oxford Vaccine Group, Department of Paediatrics, University of Oxford, and the NIHR Oxford Biomedical Research Centre, Oxford, United Kingdom; c Patan Academy of Health Sciences, Patan Hospital, Kathmandu, Nepal; d Nepal Family Development Foundation; e University of Maryland School of Medicine, Baltimore, United States; f Wasapasa Polyclinics Private Limited, Lalitpur, Kathmandu, Nepal; g Oxford University Clinical Research Unit, Vietnam; h The Department of Medicine, The University of Cambridge, UK

## Abstract

**Background:**

*Salmonella* Typhi is a major cause of fever in children in low- and middle-income countries. The recently WHO prequalified typhoid conjugate vaccine (TCV) was shown to be efficacious in a human challenge model but no efficacy trials in endemic populations have been completed.

**Methods:**

In this phase III participant- and observer-blinded randomized controlled trial in Lalitpur, Nepal, children aged 9 months to <16 years of age, were randomized 1:1 to receive either TCV or a capsular group A meningococcal conjugate vaccine (Men A) as control. The primary endpoint was blood culture-confirmed typhoid fever. Study follow-up continues for 2 years; here we present the interim analysis after 12 months of follow-up, for safety, immunogenicity and efficacy.

**Results:**

10,005 participants received TCV and 10,014 received Men A. Blood culture-confirmed typhoid fever occurred in 7 participants who received TCV and 38 receiving Men A; vaccine efficacy: 81.6% (95% CI, 58.8%, 91.8%, P<0.001). 132 SAEs occurred in the first 6 months with one (pyrexia) identified as vaccine-related. The participant remains blinded. Seroconversion (≥ four-fold rise in Vi-IgG 28 days after vaccination) was 99% in the TCV group (N=677/683) and 2% in the control group (N=8/380).

**Conclusion:**

A single dose of TCV is safe, immunogenic, and effective, and the deployment of the vaccine will reduce the burden of typhoid in high-risk populations. This new evidence of efficacy is especially timely with the recent spread of extensively drug resistant typhoid fever which threatens child health in affected regions.

**Trial registration number:**

ISRCTN43385161

## INTRODUCTION

Typhoid fever is a systemic illness caused by the *Salmonella enteric* serovar Typhi. An estimated 11 to 21 million cases of febrile illness, and 117,000 to 161,000 deaths are attributed to the disease each year^1–5^.

Typhoid fever is a major public health problem in Kathmandu, Nepal^6,7^ where *S.* Typhi accounts for up to 45% of all positive blood cultures and is the leading cause of blood-stream infections among pediatric patients ^8–10^. Typhoid is seasonal in Kathmandu, with a high season in July/August and lower incidence in winter. Annual population incidence of typhoid and paratyphoid combined has been recently estimated as 449 (95% CI, 383, 521) per 100,000 ^2^. Antibiotic-resistant S. Typhi is increasingly common in South Asia. Extensively drug-resistant (XDR) variants of S. Typhi have recently emerged in other nearby South Asian countries such as India and Bangladesh, and a large outbreak is ongoing in Pakistan, leading to a situation in which the disease in South Asian populations is becoming increasingly difficult to treat^11,12^.

The WHO recommended the use of typhoid vaccines in 2008^13^ but, vaccine-based control programs have not been widely implemented. Oral live attenuated Ty21a vaccine and Vi-polysaccharide vaccine (Vi-PS) were available but are either not tolerated (Ty21a) or poorly immunogenic in the youngest children and therefore deemed unsuitable for widespread use. A prototype TCV, Vi-rEPA (Vi conjugated to recombinant *Pseudomonas aeruginosa* exotoxin A) had over 90% efficacy in children aged 2-5 years in clinical trials in 2001 but is not available.

More recently, new generation typhoid conjugate vaccines (TCV), containing Vi polysaccharide conjugated to a tetanus-toxoid protein carrier, have become available. In a phase III safety and immunogenicity study, TCV was found to be highly immunogenic and safe in young children^14^. Furthermore, in a stringent typhoid controlled infection challenge model among adults in a non-endemic setting, TCV had a protective efficacy of 54.6% (95% CI, 26.8%, 71.8%)^15^.

In October 2017, based on these immunogenicity and human challenge study results, the WHO SAGE recommended the use of TCV over the other available typhoid vaccines in view of its improved immunological properties, suitability for use in infants and young children, and expected longer duration of protection^13^. Gavi, the Vaccine Alliance, also approved a funding window for 2019-2020 to support the introduction of TCVs in developing countries. To aid Gavi-eligible countries to accelerate the introduction of TCVs, the Typhoid Vaccine Acceleration Consortium (TyVAC) was formed^16^. We conducted the first individually randomized phase III trial of the efficacy of TCV in an endemic population, to inform vaccine implementation strategies. Herein, we report the interim results of this trial after one-year of follow-up.

## METHODS

### Study Design and Participants

A phase III, participant- and observer-blind randomized controlled trial was conducted in Lalitpur Metropolitan City of Kathmandu Valley, Nepal. Full methodology has been previously described ^17,18^. Briefly, children aged 9 months to <16 years living in the study catchment area, who were in good health at the time of enrolment, and whose parents/ legal guardian were willing and competent to provide informed consent were eligible to participate in the study. The lower age limit of 9 months was chosen to align with the potential future programmatic use of TCV given with measles vaccine at 9 months of age.

The study (ISRCTN43385161, https://doi.org/10.1186/ISRCTN43385161) was approved by the Oxford Tropical Research Ethics Committee (OxTREC 15–17) and the Nepal Health Research Council (Ref. no. 170/2017).

### Vaccines

Vi polysaccharide-tetanus toxoid conjugate vaccine (TCV, Typbar-TCV Bharat-Biotech, Hyderabad, India) containing 25 µg of Vi-polysaccharide per 0·5 mL dose was used as the trial vaccine for all age groups. Meningococcal capsular Group A conjugate vaccine (MenA; MenAfriVac, Serum Institute of India PVT Ltd) was the control vaccine (see supplementary file).

### Randomization and Blinding

Participants received either TCV or the control vaccine using 1:1 stratified block randomization with block sizes randomly varying from 2-6. Stratification was done by age (9 months to ≥5 years old or >5 years old to <16 years). Participants were randomized after consent and general examination using a bespoke randomization application loaded on an electronic tablet device.

A sub-set of children were further randomized on a 2:1 basis (1000 TCV: 500 control) to have blood drawn for immunogenicity.

Parents, guardians, participants, clinicians, and trial staff were blinded to vaccine allocation. Only the unblinded vaccinating staff were aware of the vaccine given and were not subsequently involved in participant follow-up.

### Outcomes: Assessment of Vaccine Efficacy

Blood cultures were taken from any study participant with ≥2 days of self-reported fever AND/OR a temperature of ≥ 38°C presenting to Patan hospital or 18 community-based study fever clinics. Trained physicians attended to patients, and consent was obtained for blood culture. Three-monthly follow-up phone calls were used to capture additional possible typhoid fever cases in participants who attended non-study facilities. Where available, medical records were reviewed to capture blood culture-confirmed typhoid diagnoses made at non-study hospitals and clinics. Self-treated typhoid cases, cases treated but without a blood culture taken, and cases not reported to the study team will not be captured in these study data.

The primary outcome was blood culture-confirmed typhoid fever.

### Assessment of Safety

Participants were observed at the vaccination site for at least 20 minutes after the vaccine was administered. All participants were given a diary to capture local and systemic adverse events. Participants' parents/guardians were then contacted by telephone at Day 7, to record any vaccine-related adverse events and all serious adverse events (SAEs). SAEs continue to be captured through ongoing three-monthly follow-up calls and visits.

### Immunogenicity

Anti-Vi IgG titres were measured from plasma samples collected at Day 0 and Day 28, at the Oxford Vaccine Group Laboratory, University of Oxford, using a commercial ELISA kit (VaccZyme, The Binding Site, Birmingham, UK) according to the manufacturer's instructions. Further blood samples will be collected at 18 months and 2 years of follow-up.

### Interim Analysis

The target sample size for the study was 20,000 children (see supplementary files for further details).

Over the two-year trial follow-up period 45 cases of typhoid fever were expected if the assumptions underlying the sample size held true (see supplementary files for further details). While this was originally designed as a two-year study, given the public health significance of the results, an interim analysis was planned after at least one year of follow-up had been completed, if 45 cases were observed by this time. The interim analysis therefore has full statistical power for the primary outcome. The protocol was amended to include the interim analysis when it became clear that the 45 cases may be reached before 2 years of follow-up. The interim analysis was agreed by the international data safety and monitoring board on August 1^st^, 2018, approximately 9 months into the study and received ethical approval. Study participants and staff were not unblinded as part of the interim analysis and follow-up continues.

### Statistical Analysis

The primary analysis of blood culture-confirmed typhoid fever included only those cases that occurred at least 14 days after vaccination. Additionally, secondary outcomes reported in this interim analysis include adverse events within the first 7 days after vaccination, SAEs within 6 months of vaccination and immunogenicity in the first 28 days. Full analysis of all study outcomes will be reported at the end of the study.

For the interim analysis of the primary outcome, the incidence of typhoid fever was estimated as the number of cases divided by the total number of person-years of follow-up. Vaccine efficacy (VE) was calculated as (1 – IRR) x 100%, where IRR is the incidence rate ratio (the ratio of the incidence in the TCV arm compared to the control arm).

All p-values were 2-sided; a p value < 0.05 was considered significant in efficacy assessment. Serious adverse events, local and systemic vaccine reactions, and baseline characteristics were not compared statistically.

The cumulative incidence of typhoid fever is presented using the Kaplan-Meier method. A detailed statistical analysis plan covering all analyses was agreed and signed by investigators prior to unblinding of study data for analysis and further details are included in the supplementary files.

### Author Contributions

Study design and co-ordination: AJP, KMN, MV, KTN, SS, BuBa, MS, DP, RCJ, NS, SB; Data collection and management: AA, BiBa, MG, SK, OM, YF, ST, and the TyVAC Nepal Study Team (see supplementary file); Laboratory analysis: JC, JH, SD, AK; Data analysis: MV, XL; MV vouches for the data and analysis; Final decision to submit for publication: AJP; MS prepared the first draft manuscript, which was reviewed and approved by all authors.

## RESULTS

### Study Participants

From November 20, 2017 to April 9, 2018, 20119 children were screened, and 20,019 participants were randomized to receive the TCV or control vaccine (Figure S1). The baseline characteristics were similar in the TCV recipients and the control vaccine recipients (Table 1).

**Table 1:** Baseline characteristics of randomised participants

Characteristics	TCV *(N=10,005)*	Men A *(N=10 014)*	Total *(N= 20019)*
Gender			
*Male N (%)*	5106 (51.0%)	5158 (51.5%)	10264 (51.3%)
Age at enrolment (years)			
*Mean (SD)*	7.9 (4.1)	7.8 (4.0)	7.9 (4.1)
*Median [Range]*	7.7 [0.8 – 16.1]	7.7 [0.7 – 16.1]	7.7 [0.7 – 16.1] *
*< 5 years N (%)*	2907 (29.1%)	2905 (29.0%)	
*≥ 5 years N (%)*	7098 (70.9%)	7109 (71.0%)	
Self-reported medical history of Typhoid fever**			
*N (%)*	345 (3.5%)	395 (4.0%)	740 (3.7%)

* 6 participants are outside the age range for eligibility (9 months to 15 years + 364 days)

** Self-reported history of typhoid infection prior to the beginning of the study, reported at baseline intake

TCV = Typhoid Conjugate Vaccine. Men A = Group A meningococcal vaccine (control)

### Vaccine Efficacy

Between Dec 6, 2017 and March 9, 2019, 46 cases of blood culture-confirmed typhoid fever were recorded. One case occurred within 2 weeks of vaccination and was excluded from analyses. All cases recovered; 5 were admitted to hospital (TCV: 2, Control: 3).

**Figure 1:**
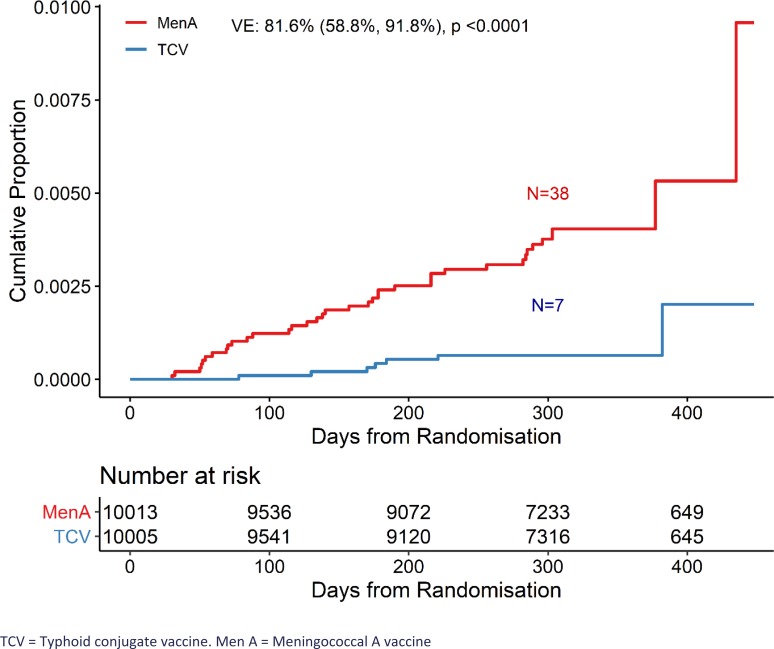
Kaplan-Meier cumulative incidence of blood culture-positive typhoid fever by randomised vaccine group

Blood culture-confirmed typhoid fever was diagnosed in 0.07% (n=7 of 10,005) of participants in the TCV group and 0.38% (n=38 of 10,013) in the control group. The protective efficacy of TCV was 81.6% (95% CI, 58.8%, 91.8%, P<0.001) (Figure 1, Table 2).

**Table 2:** Occurrence of blood culture-confirmed typhoid fever and protective efficacy of typhoid conjugate vaccine

Outcome	TCV (N=10005)	Incidence per 100,000 person-years (95% CI)	Men A (N=10014)	Incidence per 100,000 person-years (95% CI)	Vaccine Efficacy (95% CI)	p value (Log-rank)
Person-years of follow-up ^a^	8903		8885			

Blood culture-confirmed typhoid fever in first 14 days after vaccination			1			
**Blood culture-confirmed typhoid fever after 14 days ^b^**	**7**	**79 (37, 165)**	**38**	**428 (311, 588)**	**81.6% (58.8%, 91.8%)**	**<0.001**
*Detected through fever clinics*	*5*		*27*			
*Detected through active follow-up and medical record review*	*2*		*11*			
Blood culture-confirmed typhoid fever in those with at least 3 days of fever prior to blood culture (fever clinics) ^c^	3	34 (11, 105)	20	226 (146, 350)	85.1% (49.7%, 95.6%)	<0.001

a Participants with no follow-up contact contribute half a day follow-up in calculations. Participants who move away from Lalitpur no longer contribute to person-years of follow-up time

b For all reported culture positive cases reported from medical records review, isolates were checked, when available, to reconfirm diagnostic results.

c From fever clinic cases only. Data not available from cases detected through medical records review.

TCV = Typhoid conjugate vaccine. Men A = Group A meningococcal vaccine

There were 23 cases of blood culture-confirmed typhoid fever in those presenting to fever clinics with at least 3 days of fever prior to their blood draw for culture; the WHO recommended threshold for blood cultures in typhoid surveillance programs^19^. Vaccine efficacy in those with at least 3 days of fever was similar to the overall estimate: 85.1% (95%CI, 49.7%, 95.6%).

### Immunogenicity

1343 participants provided at least one sample for immunogenicity analysis. At baseline, 268 (31.6%) participants in the TCV group and 122 (26.5%) participants in the Men A group had detectable Vi-IgG antibody levels. The geometric mean titre of anti-Vi antibody at day 28 was 2038 EU/mL (95% CI, 1905, 2180) for the TCV group and 7.0 EU/mL (95% CI, 6.2, 7.9) for the Men A group (p<0.001). Seroconversion (≥ four-fold rise in antibody titre 28 days after vaccination) was 99% in the TCV group and 2% in the control group (Table 3).

**Table 3:** Vi-IgG levels at baseline and 28 days after randomization in immunogenicity cohort

Visit	TCV			Men A			p value*
	N	N (%) above LLD	GMC (95%CI) or Median [IQR] or %	N	N (%) above LLD	GMC (95%CI) or Median [IQR] or %
**Day 0**	849	268 (31.6%)	7.2 (6.7, 7.8) ^a^	460	122 (26.5%)	6.5 (5.9, 7.1) ^a^	
			3.7 [3.7, 13.4] ^b^			3.7 [3.7, 8.9] ^b^	0.07
**Day 28**	709	708 (99.9%)	2038 (1905, 2180) ^a^	388	112 (28.9%)	7.0 (6.2, 7.9) ^a^	
			2221 [1297, 3726] ^b^			[3.7, 10.5] ^b^	<0.001
**Both Day 0 & Day 28**	683			380			
**≥ 4-fold rise from Day 0**	677		99.1%	8		2.1%	

LLD: The lower limit of quantification of the assay (7.4 EU/mL). Values below this limit were substituted with 3.7 EU/mL for analysis. GMC = geometric mean concentration

* p value from non-parametric two-sided Wilcoxon Rank Sum Test. TCV = Typhoid conjugate vaccine. Men A = Group A meningococcal vaccine (control).

a geometric mean concentration (95% confidence interval);

b median [interquartile range]

### Reactogenicity

Adverse vaccine reactions in the first 7 days after vaccination were assessed in 18,743 (93.6%) children. 5.9% of children experienced pain at the vaccination site (TCV: 5.1%, Men A: 6.7%) which was mostly mild (92.6%). 6.7% of children reported being generally unwell (TCV: 6.4%, Men A: 7.1%). 5.2% of children had a fever (by parental self-report) in the first 7 days (TCV: 5.0%, Men A: 5.4%). Vomiting and diarrhea occurred in 1.4% and 1.8% of children respectively (Vomiting: TCV: 1.2%, Men A: 1.6%; Diarrhea: TCV: 1.7%, Men A 1.8%), and of those reporting these symptoms, 20.5% and 25.9% of instances were moderate or severe. 1.9% of children were eating less than usual (TCV: 1.8%, Men A: 1.9%). All other reactions were rare, occurring in less than 1% of children (Table S1).

### Serious Adverse Events

In the first 28 days after vaccination, 18 SAEs were reported in 17 participants; 7 participants in the TCV group and 10 in the Men A group (Tables S2 and S3). One SAE was identified as vaccine-related; a high-grade fever within 24 hours of vaccination. The participant was admitted to the local hospital and given antipyretics. The fever subsided after 12 hours, investigations were within normal limits and the participant was discharged without an alternative diagnosis. The participant remains blinded (Tables S2 and S3).

SAEs occurring in the 6 months after vaccination were reported by 121 participants who experienced 132 events. (Table S4). SAEs occurring more than once per group are summarised by MedDRA codes in Table S5. The most common SAEs were pneumonia/lower respiratory tract infection and pyrexia.

There was one death due to staphylococcal sepsis, occurring 7 months after vaccination, deemed unrelated to vaccination (see Supplementary file).

## DISCUSSION

This is the first large-scale field trial to assess the efficacy of a WHO prequalified typhoid conjugate vaccine in children in an endemic setting and shows that a single dose of TCV is safe, immunogenic, efficacious, and has the potential to save thousands of lives. Incidence was 428 per 100,000 in our control group, confirming the high burden of disease in children in this setting.

Large-scale vaccination strategies using TCV can potentially reduce the burden of enteric fever, an important goal given the global increase in antimicrobial resistance. The rise in extensively drug resistant (XDR) typhoid severely limits treatment options. Over 5000 cases of XDR typhoid have been reported in Pakistan since the outbreak began in 2016, with cases also being reported in travelers returning from Pakistan. Deployment of the vaccine in Pakistan, as is being done, and beyond, is of paramount importance to curb the spread of the drug resistant strain regionally and transcontinentally.

A single dose of TCV resulted in a reduction in typhoid fever by 81.6% in children in our study. This protective efficacy is higher than that of Vi-PS which was estimated to have 35% to 65% efficacy in trials in Pakistan and India respectively ^20,21^, and higher than live attenuated oral typhoid vaccines^22^. The results are similar to the 91.1% (95% CI, 77.1%, 96.6%) efficacy seen with two doses of Vi-rEPA in Vietnam in 1997^23^. The results are also consistent with the seroefficacy estimates (85%, 95% CI 80%, 88%) of TCV extrapolated from serological responses in the phase 3 trial in India^24^.The vaccine efficacy was 54.6% (95%CI, 26.8%, 71.8%) in a human challenge study conducted in Oxford^15^. However, the challenge model used a composite definition of typhoid fever that included self-resolving asymptomatic bacteraemia not detected in the field, adults rather than children, and a probable high challenge dose (following neutralization of gastric acid), which could provide some explanation as to why vaccine efficacy was lower compared with our results.

TCV is highly immunogenic, eliciting a strong antibody response one month after vaccination. This is consistent with previous findings in immunogenicity trials ^14,15,23^. Immunogenicity trials in children and adults in India reported seroconversion rates of over 90% across different age strata at day 42 post-vaccination compared to baseline titres and a four-fold rise in anti Vi- antibody titre occurred 2 to 5 times more often in the TCV group in comparison with the Vi-PS group^14^. The Vi-rEPA study reported that Vi-IgG increased by a factor of more than 575 (P<0.001) four weeks after administration of the conjugate Vi-rEPA vaccine, although using a different assay^23^. Conjugate vaccines are T-cell dependent and are expected to provide long-term protection as demonstrated with the Vi-rEPA vaccine unlike the protection provided by polysaccharide vaccines which generally last for only 2-3 years^25^.

TCV was safe and clinically acceptable in this study. Our data on reactogenicity to the vaccine are consistent with those from the phase III trial in India ^14^, and the human challenge model study^15^. In our study, one SAE was deemed to be a vaccine-related fever without any alternative diagnosis, but remains blinded to group allocation. Reported adverse events were similar for both TCV and the control vaccine, indicating an acceptable safety profile in comparison with another widely used conjugate vaccine. These data were part of a package reviewed by the WHO Global Advisory Committee on Vaccine Safety in December 2018, leading to an endorsement of the safety of this vaccine^26^.

These results provide strong evidence that TCV can play an important role in the control of typhoid fever in endemic settings. TCV is highly cost-effective in high transmission settings and should be taken into account in country decision-making ^27^. However, further data are still required to demonstrate vaccine efficacy in the medium- and long-term, the indirect effect and herd immunity achieved from large-scale vaccination, and the effectiveness in different age groups and populations. The full analysis of data from this trial, as well as data from on-going trials in Malawi and Bangladesh, will be available within the next two years to address these outstanding questions^28,29^.

Our findings uphold the WHO's recent recommendations to use TCV to control typhoid in high burden settings through immunization of children from 9 months to 15 years of age^5^. Inclusion of the conjugate vaccine in routine immunization schedules in high burden countries could prevent a large burden of a disease that has been disproportionately affecting children.

## References

[1] Antillón M, Warren JL, Crawford FW, et al. The burden of typhoid fever in low- and middle-income countries: A meta-regression approach. PLoS Negl Trop Dis 2017;11(2):1–21. 10.1371/journal.pntd.0005376 PMC5344533 28241011

[2] Stanaway JD, Reiner RC, Blacker BF, et al. The global burden of typhoid and paratyphoid fevers: a systematic analysis for the Global Burden of Disease Study 2017. Lancet Infect Dis 2019;19(4):369–81. 30792131 10.1016/S1473-3099(18)30685-6 PMC6437314

[3] Buckle GC, Walker CLF, Black RE Typhoid fever and paratyphoid fever: Systematic review to estimate global morbidity and mortality for 2010. J Glob Health 2012;2(1):010401. 23198130 10.7189/jogh.02.010401 PMC3484760

[4] Mogasale Vittal, Maskery Brian Burden of typhoid fever in low-inocme and middle-income cuntries: a systematic, literature-based update with risk-factor adjustment. Lancet Glob Heal 2014;2:570–80. 10.1016/S2214-109X(14)70301-8 25304633

[5] World Health Organisation Typhoid vaccines: WHO position paper, March 2018 – Recommendations. Vaccine 2019;37(2):214–6. 29661581 10.1016/j.vaccine.2018.04.022

[6] Karkey A, Aryjal A, Basnyat B, Baker S Mini-Review Article Kathmandu , Nepal : Still an enteric fever capital of the world. J Infect Dev Ctries 2005;4–8. 10.3855/jidc.162 19745524

[7] Karkey A, Arjyal A, Anders KL, Boni MF, Dongol S The Burden and Characteristics of Enteric Fever at a Healthcare Facility in a Densely Populated Area of Kathmandu. PLoS One 2010;5(11):e13988. 21085575 10.1371/journal.pone.0013988 PMC2981554

[8] Pradhan R, Shrestha U, Gautam SC, et al. Bloodstream Infection among Children Presenting to a General Hospital Outpatient Clinic in Urban Nepal. PLoS One 2012;7(10):e47531. 23115652 10.1371/journal.pone.0047531 PMC3480362

[9] Zellweger RM, Basnyat B, Shrestha P, et al. A 23-year retrospective investigation of Salmonella Typhi and Salmonella Paratyphi isolated in a tertiary Kathmandu hospital. PLoS Negl Trop Dis 2017;11(11):1–16. 10.1371/journal.pntd.0006051 PMC5720835 29176850

[10] Kelly DF, Thorson S, Maskey M, et al. The burden of vaccine-preventable invasive bacterial infections and pneumonia in children admitted to hospital in urban Nepal. Int J Infect Dis 2011;15(1):e17–23. 21123100 10.1016/j.ijid.2010.05.021

[11] Andrews JR, Qamar FN, Charles RC, Ryan ET Extensively Drug-Resistant Typhoid — Are Conjugate Vaccines Arriving Just in Time? New Engl J Med 2018;379(16):1493–5. 30332569 10.1056/NEJMp1803926

[12] Klemm EJ, Shakoor S, Page AJ, et al. Emergence of an Extensively Drug-Resistant Salmonella enterica Serovar Typhi Clone Harboring a Promiscuous Plasmid Encoding Resistance to Fluoroquinolones and Third-Generation Cephalosporins. MBio 2018;9(1):1–10. 10.1128/mBio.00105-18 PMC5821095 29463654

[13] SAGE Working Group on Typhoid Vaccines and the WHO Secretariat Background paper to SAGE on typhoid vaccine policy recommendations. Strateg Advis Gr Expert Immun - 17-19 Oct 2017, WHO HQ, Geneva Switz 2017;(9):105–204.

[14] Mohan VK, Varanasi V, Singh A, et al. Safety and Immunogenicity of a Vi Polysaccharide-Tetanus Toxoid Conjugate Vaccine (Typbar-TCV) in Healthy Infants, Children, and Adults in Typhoid Endemic Areas: A Multicenter, 2-Cohort, Open-Label, Double-Blind, Randomized Controlled Phase 3 Study. Clin Infect Dis 2015;61(3):393–402. 25870324 10.1093/cid/civ295

[15] Jin C, Gibani MM, Moore M, et al. Efficacy and immunogenicity of a Vi-tetanus toxoid conjugate vaccine in the prevention of typhoid fever using a controlled human infection model of Salmonella Typhi: a randomised controlled, phase 2b trial. Lancet 2017;390(10111):2472–80. 28965718 10.1016/S0140-6736(17)32149-9 PMC5720597

[16] Meiring JE, Gibani M, Basnyat B, et al. The Typhoid Vaccine Acceleration Consortium (TyVAC): Vaccine effectiveness study designs: Accelerating the introduction of typhoid conjugate vaccines and reducing the global burden of enteric fever. Report from a meeting held on 26–27 October 2016, Oxford. J Vaccine 2017;35(38):5081–8. 10.1016/j.vaccine.2017.08.001 28802757

[17] Theiss-Nyland K, Shakya M, Colin-Jones R, et al. Assessing the Impact of a Vi-polysaccharide Conjugate Vaccine in Preventing Typhoid Infections Among Nepalese Children: A Protocol for a Phase III, Randomized Control Trial. Clin Infect Dis 2019;68(Supplement 2):S67–73. 30845329 10.1093/cid/ciy1106 PMC6405280

[18] Colin-Jones R, Shakya M, Voysey M, et al. Logistics of Implementing a Large-scale Typhoid Vaccine Trial in Kathmandu, Nepal. Clin Infect Dis 2019;68(Supplement 2):S138–45. 30845335 10.1093/cid/ciy1125 PMC6405269

[19] Surveillance standards for vaccine-preventable diseases, second edition Geneva: World Health Organization; 2018 Licence: CC BY-NC-SA 3.0 IGO.;

[20] Sur D, Ochiai RL, Bhattacharya SK, et al. A Cluster-Randomized Effectiveness Trial of Vi Typhoid Vaccine in India. N Engl J Med 2009;361(4):335–44. 19625715 10.1056/NEJMoa0807521

[21] Khan MI, Soofi SB, Ochiai RL, et al. Effectiveness of Vi capsular polysaccharide typhoid vaccine among children: A cluster randomized trial in Karachi, Pakistan. Vaccine 2012;30(36):5389–95. 22721899 10.1016/j.vaccine.2012.06.015

[22] Simanjuntak CH, Totosudirjo H, Haryanto P, et al. Oral immunisation against typhoid fever in Indonesia with Ty21a vaccine. Lancet 1991;338(8774):1055–9. 1681365 10.1016/0140-6736(91)91910-m

[23] Lin FYC, Ho VA, Khiem HB, et al. The efficacy of a Salmonella typhi Vi conjugate vaccine in two-to-five-year-old children. N Engl J Med 2001;344(17):1263–9. 11320385 10.1056/NEJM200104263441701

[24] Voysey M, Pollard AJ Seroefficacy of Vi Polysaccharide–Tetanus Toxoid Typhoid Conjugate Vaccine (Typbar TCV). Clin Infect Dis 2018;67(1):18–24. 29351594 10.1093/cid/cix1145

[25] Yang HH, Wu CG, Xie GZ, et al. Efficacy trial of Vi polysaccharide vaccine against typhoid fever in south-western China. Bull World Health Organ 2001;79(7):625–31. 11477965 PMC2566475

[26] World Health Organization Weekly epidemiological record - Global Advisory Committee on Vaccine Safety 5 - 6 December 2018. 2019.

[27] Bilcke J, Antillón M, Pieters Z, et al. Cost-effectiveness of routine and campaign use of typhoid Vi-conjugate vaccine in Gavi-eligible countries: a modelling study. Lancet Infect Dis 2019;19(7):728–39. 31130329 10.1016/S1473-3099(18)30804-1 PMC6595249

[28] Meiring JE, Laurens MB, Patel P, et al. Typhoid Vaccine Acceleration Consortium Malawi: A Phase III, Randomized, Double-blind, Controlled Trial of the Clinical Efficacy of Typhoid Conjugate Vaccine Among Children in Blantyre, Malawi. Clin Infect Dis - Suppl Artic 2019;68(Suppl 2):S51–8. 10.1093/cid/ciy1103 PMC6405268 30845320

[29] Theiss-Nyland K, Qadri F, Colin-Jones R, et al. Assessing the Impact of a Vi-polysaccharide Conjugate Vaccine in Preventing Typhoid Infection Among Bangladeshi Children: A Protocol for a Phase IIIb Trial. Clin Infect Dis 2019;68(Supplement 2):S74–82. 30845333 10.1093/cid/ciy1107 PMC6405281

