# Supplementary material for: Efficacy of Typhoid Conjugate Vaccine in Nepal: An Interim Analysis of a Participant-and Observer-Blinded Randomized Phase III Trial
Source: N Engl J Med. Author manuscript; Available in PMC 2019 Dec 5. (PMC6785806; doi:10.1056/NEJMoa1905047)
Supplement: Supplementary file 1 [file NEJM-2019-1905047-s1.pdf]

# Supplementary materials

## Contents

1.1 Acknowledgments .......... 2

1.2 Vaccines administered .......... 2

1.3 Samples size considerations .......... 2

1.4 Deaths .......... 3

2 Supplementary Figures and Tables .......... 4

Figure S1 Trial flow diagram .......... 4

Table S1 Self-Reported solicited adverse reactions within 7 days of vaccination .......... 5

Table S2 Serious adverse events (SAEs) occurring within 28 days of vaccination .......... 7

Table S3 MedDRA coded serious adverse events (SAEs) occurring within 28 days of vaccination .......... 7

Table S4 Serious adverse events (SAEs) within 6 months of vaccination .......... 8

Table S5 MedDRA coded serious adverse events (SAEs) occurring within 6 months of vaccination .......... 9

## 1.1 Acknowledgments

The authors acknowledge the valuable contributions made by the TyVAC Nepal Study Team: Aseem Poudel[a], Baibhav Mallik[a], Deepak Ghimire[a], Mamata Yadav[a], Roshan Subedi[a], Sachita Baniya[a], Sunil Thapa[a], Naheeda Haque[a], Aashata Dahal[a], Archana Maharjan[a], Pallavi Gurung[a], Kristina Joshi[a], James Meiring[b], Kopila Lama[c], Jasmine Maharjan[c], Sima Gurung[c], Nissa Humagain[c], Grishu Shrestha[c], Bhola Prasad Koirala[c], Pratikshaya Kisiju[c], Kabita Pathak[c], Surendra Adhikari[c], Anjali Joshi[c], Ashesh Chhetri[c], Alina Maharjan[c], Tan Trinh Van[d].

[a]Oxford University Clinical Research Unit, Patan Academy of Health Sciences, Kathmandu, Nepal

[b]Oxford Vaccine Group, Department of Paediatrics, University of Oxford, and the NIHR Oxford Biomedical Research Centre, Oxford, United Kingdom

[c]Nepal Family Development Foundation

[d] Oxford University Clinical Research Unit, Vietnam

## 1.2 Vaccines administered

The trial vaccine, Vi polysaccharide-tetanus toxoid conjugate vaccine (TCV, Typbar –TCV Bharat-Biotech, Hyderabad, India) was available as a 2.5ml 5-dose vial, with each 0.5 ml vaccine dose containing purified Vi-capsular polysaccharide of S. Typhi Ty2 conjugated to 25 mcg Tetanus Toxoid. Meningococcal capsular Group A conjugate vaccine (Men A; MenAfriVac, Serum Institute of India PVT Ltd) was used as the control vaccine. The Men A vaccine was produced in two formulations; a standard 10 mcg/0.5 ml dose for individuals ≥ 1 year of age and a 5 mcg/ 0.5ml dose for children 9 – 24 months of age. In the trial, children under 1 year of age were given the 5 mcg/ 0.5ml dose. The control vaccine was provided in a 10-dose vial of active vaccine, each vial containing a lyophilized powder of meningococcal group A polysaccharide conjugated to tetanus toxoid protein; and, 10 dose ampoules of diluent. Both vaccines were stored at 2 to 8 degrees C and cold chain maintained in vaccination clinics. The diluents were stored at room temperature. Opened vaccine vials were discarded 6 hours after opening. All vaccines were given intramuscularly.

## 1.3 Samples size assumptions

Sample size calculations were based on the 80% power and 5% alpha with the additional following assumptions:

1. An overall incidence of typhoid fever of 85 cases per year, per 100,000 persons in the entire population, with higher incidence rates in children under 16 years.
2. Age-specific distribution of typhoid cases in Kathmandu determined from published estimates and from unpublished site-specific surveillance data.

3. A direct effect of vaccination of 75% and an indirect effect of 25% based on mathematical modelling.
4. 25% loss to follow-up per year due to participants moving out of the area, based on current surveillance data from Patan.

With the above assumptions the required sample size was 17,395 children. To allow for variation in these assumptions, the total target sample size was increased 20,000 children to increase power (10,000 in each vaccination arm).

## 1.4 Deaths

A 38-month-old presented with fever, cough, fast and noisy breathing, and increasing lethargy. He was found to be in septic shock with bilateral crepitations on auscultation, and his blood culture revealed a heavy growth of *Staphylococcus aureus*. He was mechanically ventilated, required inotropic support and consequently developed multiple organ dysfunction.

## 2 Supplementary Figures and Tables

**Figure S1 Trial flow diagram**

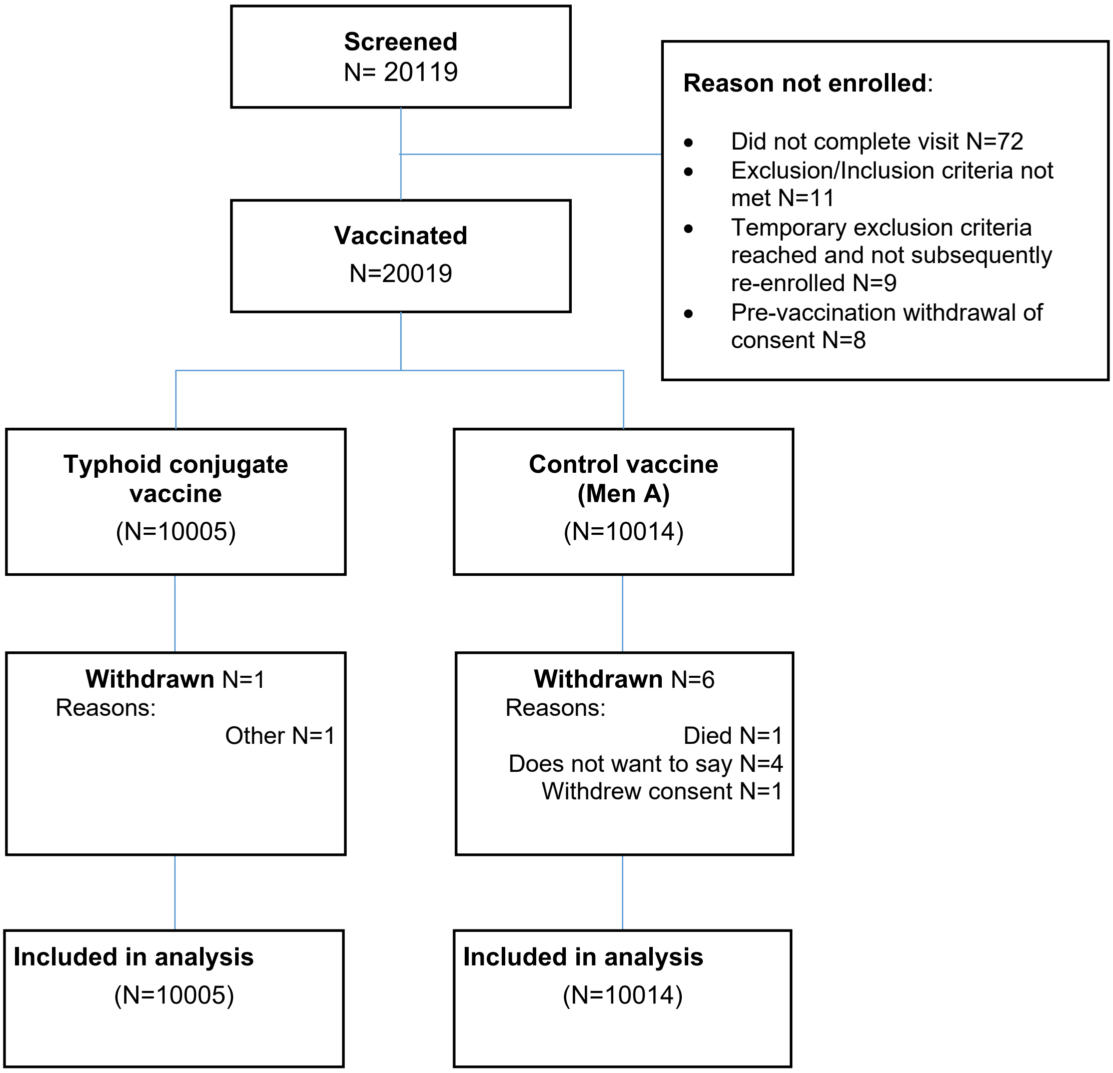

**Table S1 Self-Reported solicited adverse reactions within 7 days of vaccination**

| | *TCV* | | *Men A* | | All | |
|---|---|---|---|---|---|---|
| | *N* | *%* | *N* | *%* | *N* | *%* |
| ***Pain*** | | | | | | |
| *None* | 8898 | 94.86 | 8732 | 93.26 | 17630 | 94.06 |
| *Mild* | 455 | 4.85 | 575 | 6.14 | 1030 | 5.50 |
| *Moderate* | 26 | 0.28 | 53 | 0.57 | 79 | 0.42 |
| *Severe* | 1 | 0.01 | 3 | 0.03 | 4 | 0.02 |
| ***Swelling*** | | | | | | |
| *None* | 9322 | 99.38 | 9273 | 99.04 | 18595 | 99.21 |
| *Mild* | 53 | 0.57 | 81 | 0.87 | 134 | 0.72 |
| *Moderate* | 5 | 0.05 | 9 | 0.10 | 14 | 0.08 |
| ***Redness*** | | | | | | |
| *None* | 9369 | 99.88 | 9345 | 99.81 | 18714 | 99.85 |
| *Mild* | 10 | 0.11 | 14 | 0.15 | 24 | 0.13 |
| *Moderate* | 1 | 0.01 | 4 | 0.04 | 5 | 0.03 |
| ***Self-reported Fever**** | | | | | | |
| *No* | 8907 | 94.96 | 8861 | 94.64 | 17768 | 94.80 |
| *Yes* | 473 | 5.04 | 502 | 5.36 | 975 | 5.20 |
| ***Vomiting*** | | | | | | |
| *None* | 9272 | 98.85 | 9213 | 98.40 | 18485 | 98.62 |
| *Mild* | 84 | 0.90 | 121 | 1.29 | 205 | 1.09 |
| *Moderate* | 23 | 0.25 | 25 | 0.27 | 48 | 0.26 |
| *Severe* | 1 | 0.01 | 4 | 0.04 | 5 | 0.03 |
| ***Diarrhoea*** | | | | | | |
| *None* | 9216 | 98.25 | 9195 | 98.21 | 18411 | 98.23 |
| *Mild* | 126 | 1.34 | 120 | 1.28 | 246 | 1.31 |
| *Moderate* | 37 | 0.39 | 44 | 0.47 | 81 | 0.43 |
| *Severe* | 1 | 0.01 | 4 | 0.04 | 5 | 0.03 |
| ***Less active*** | | | | | | |
| *None* | 9326 | 99.42 | 9284 | 99.16 | 18610 | 99.29 |
| *Mild* | 43 | 0.46 | 69 | 0.74 | 112 | 0.60 |
| *Moderate* | 11 | 0.12 | 8 | 0.09 | 19 | 0.10 |
| *Severe* | 0 | | 2 | 0.02 | 2 | 0.01 |
| ***Cried persistently*** | | | | | | |
| *None* | 9362 | 99.81 | 9328 | 99.63 | 18690 | 99.72 |
| *Mild* | 15 | 0.16 | 27 | 0.29 | 42 | 0.22 |
| *Moderate* | 3 | 0.03 | 6 | 0.06 | 9 | 0.05 |
| *Severe* | 0 | | 2 | 0.02 | 2 | 0.01 |
| ***Eating less*** | | | | | | |

| | | | | | | |
|---|---|---|---|---|---|---|
| *None* | 9207 | 98.16 | 9187 | 98.12 | 18394 | 98.14 |
| *Mild* | 144 | 1.54 | 144 | 1.54 | 288 | 1.54 |
| *Moderate* | 29 | 0.31 | 29 | 0.31 | 58 | 0.31 |
| *Severe* | 0 | | 3 | 0.03 | 3 | 0.02 |
| ***More irritable*** | | | | | | |
| *None* | 9348 | 99.66 | 9318 | 99.52 | 18666 | 99.59 |
| *Mild* | 26 | 0.28 | 40 | 0.43 | 66 | 0.35 |
| *Moderate* | 6 | 0.06 | 4 | 0.04 | 10 | 0.05 |
| *Severe* | 0 | | 1 | 0.01 | 1 | 0.01 |
| ***Generally Unwell*** | | | | | | |
| *Well* | 8780 | 93.60 | 8700 | 92.92 | 17480 | 93.26 |
| *Unwell* | 600 | 6.40 | 663 | 7.08 | 1263 | 6.74 |
| *All* | 9380 | 100.00 | 9363 | 100.00 | 18743 | 100.00 |

TCV = Typhoid conjugate vaccine. MenA = Group A meningococcal vaccine (control). * Self-reported fever or feeling feverish. No temperatures readings were taken.

**Table S2 Serious adverse events (SAEs) occurring within 28 days of vaccination**

| Characteristics | TCV (N=10005) | Men A (N=10014) | Total (N=20019) |
|---|---|---|---|
| **Number of participants with SAEs** | 7 | 10 | 17 |
| **Number of SAEs** | 7 | 11 | 18 |
| **Severity** | | | |
| Mild | 1 | 1 | 2 |
| Moderate | 5 | 7 | 12 |
| Severe | 1 | 3 | 4 |

SAEs were defined as outcomes requiring hospitalization, were life-threatening or resulted in disability, incapacity or death. SAEs were observed by the investigator, members of the study team or reported by parent or guardians by telephone contact.

Severity was defined as the intensity of the specific event as reported by parents or guardian.

One SAE was deemed related to vaccine but remains blinded until the end of the trial and is therefore not reported herein.

TCV = Typhoid conjugate vaccine. Men A = Group A meningococcal vaccine (control)

**Table S3 MedDRA coded serious adverse events (SAEs) occurring within 28 days of vaccination**

| MedDRA System Organ Class and Preferred Terms | | TCV | Men A | All |
|---|---|---|---|---|
| **Gastrointestinal disorders** | Gastrointestinal disorders | 2 | 2 | 4 |
| **General disorders and administration site conditions** | Pyrexia | 1 | 3 | 4 |
| **Infections and infestations** | Gastroenteritis | 1 | 2 | 3 |
| | Pneumonia/LRTI | 3 | 3 | 6 |
| | URTI /Viral infection | 1 | 1 | 2 |
| **Metabolism and nutrition disorders** | Dehydration | 1 | 1 | 2 |
| **Nervous system disorders** | Febrile convulsion | 2 | 2 | 4 |

*Only events occurring more than once are shown in order to maintain blinding of participants experiencing rare events.

LRTI = Lower respiratory tract infection, URTI = upper respiratory tract infection, TCV = Typhoid conjugate vaccine. Men A = Group A meningococcal vaccine (control)

**Table S4 Serious adverse events (SAEs) within 6 months of vaccination**

| Characteristics | TCV (N=10005) | Men A (N=10014) | Total (N=20019) |
|---|---|---|---|
| **Number of participants with SAEs** | 58 | 63 | 121 |
| **Number of SAEs** | 61 | 71 | 132 |
| **Severity** | | | |
| Mild | 1 | 4 | 5 |
| Moderate | 59 | 63 | 122 |
| Severe | 1 | 4 | 5 |

SAEs were defined as outcomes requiring hospitalization, were life-threatening or resulted in disability, incapacity or death. SAEs were observed by the investigator, members of the study team or reported by parent or guardians by telephone contact.

Severity was defined as the intensity of the specific event as reported by parents or guardian.

One SAE was deemed related to vaccine but remains blinded until the end of the trial and is therefore not reported herein.

TCV = Typhoid conjugate vaccine. Men A = Group A meningococcal vaccine (control)

**Table S5 MedDRA coded serious adverse events (SAEs) occurring within 6 months of vaccination**

| | | TCV | Men A | All |
|---|---|---|---|---|
| **Gastrointestinal disorders** | Gastrointestinal disorders | 9 | 9 | 18 |
| **General disorders and administration site conditions** | Pyrexia | 11 | 11 | 22 |
| **Infections and infestations** | Appendicitis/appendicectomy | 2 | 1 | 3 |
| | Bacterial infection | 1 | 1 | 2 |
| | Gastroenteritis | 7 | 7 | 14 |
| | Pneumonia/LRTI | 21 | 11 | 32 |
| | Typhoid fever | 1 | 2 | 3 |
| | URTI | 5 | 7 | 12 |
| | Urinary tract infection | 3 | 7 | 10 |
| **Metabolism and nutrition disorders** | Dehydration | 5 | 4 | 9 |
| | Hyponatraemia | 1 | 2 | 3 |
| **Musculoskeletal and connective tissue disorders** | Musculoskeletal and connective tissue disorders | 1 | 2 | 3 |
| **Nervous system disorders** | Febrile convulsion | 5 | 8 | 13 |
| | Seizure | 1 | 2 | 3 |
| **Respiratory, thoracic and mediastinal disorders** | Bronchospasm | 3 | 5 | 8 |
| **Skin and subcutaneous tissue disorders** | Skin and subcutaneous tissue disorders | 2 | 1 | 3 |

*Only events occurring more than once are shown in order to maintain blinding of participants experiencing rare events.

LRTI = Lower respiratory tract infection, URTI = upper respiratory tract infection, TCV = Typhoid conjugate vaccine. Men A = Group A meningococcal vaccine (control)
